# Streamlining genetic testing by standardizing its report formats and exchange among all stakeholders

**DOI:** 10.1186/1878-5085-5-S1-A61

**Published:** 2014-02-11

**Authors:** Amnon Shabo

**Affiliations:** 1 IBM Haifa Research Lab, Israel

## Scientific objectives

A growing base of genetic testings is now available for clinicians and its results could improve the quality of care and its outcomes. In addition, these testings are also helpful for predictive medicine and early detection of diseases. Genetic testings could further personalize the care processes based on the patient individual genetic makeup. Genetic testing methods are diverse and span from testing for known germline mutations in the context of single-gene disorders, to full sequencing of genes in tumor tissues looking for somatic variations in cancer cells, and on to whole-exome sequencing in cases of predictive medicine or unknown diagnosis. As a consequence of that diversity and the constantly growing number of techniques yielding new result formats less familiar to clinicians, existing report formats attempt to contain rather detailed descriptions of the tests performed, but that approach makes it hard to understand the interpretations of the testing results and given recommendations. Genetic testing reports should be both human readable to its users, and at the same time also be processable to computerized algorithms computing the interpretation of the genetic testing results. Thus, it is equally important to standardize the way genetic testing interpretations are represented [1] as an inherent part of the report structure.

## Technological approaches

**Figure 1:**
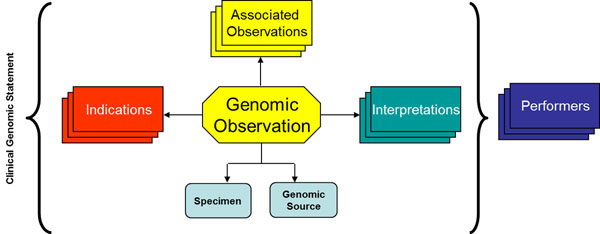
The Clinical Genomics Statement: Towards standardized language bridging the genomic and clinical worlds.

Given the above considerations, it is important to streamline the utilization of genetic testing reports by standardizing its format. The GTR (Genetic Testing Report) standard was published in February 2013 [2]. It is derived from the CDA (Clinical Document Architecture) standard [3] and further specializes the CDA clinical statement model in order to represent genotype-phenotype associations. The GTR Clinical Genomic Statement suggests a semantic representation (see figure 1) of genotype-phenotype associations, in a way that decision support applications can run reasoning algorithms without basing the inference on implicit associations where prior knowledge is needed [1].

## Results

The GTR standard can facilitate true semantic interoperability among all personalized healthcare stakeholders, e.g., genetic labs, EHR systems, patients, clinical trials and research, public health agencies, etc. The GTR is flexible enough to be further constrained for more specific usages, e.g., requirements in different countries, research vs. clinical environments, professional societies recommended-formats, specific omics technologies / methodologies, or special use cases such as tissue typing or whole-exome sequencing report.

## Outlook and expert recommendations

The GTR has been developed using MDHT (Model-Driven Health Tool) - an open source effort that is used worldwide to develop specialized standards based on UML models of common and generic standards like the CDA [4]. The MDHT also enables automatic code generation of software components that impalement the GTR and thus could facilitate the streamlining of genetic testing report utilization, as it is easier to develop or upgrade healthcare information systems to support the GTR.
